# The pCREB/BDNF Pathway in the Hippocampus Is Involved in the Therapeutic Effect of Selective 5-HT Reuptake Inhibitors in Adult Male Rats Exposed to Blast Traumatic Brain Injury

**DOI:** 10.3390/brainsci15030236

**Published:** 2025-02-24

**Authors:** Xiaolin Fan, Hong Wang, Xiaoqiang Lv, Qi Wang, Boya Yu, Xiao Li, Liang Li, Yuhao Zhang, Ning Ma, Qing Lu, Airong Qian, Junhong Gao

**Affiliations:** 1 Laboratory for Bone Metabolism, Xi’an Key Laboratory of Special Medicine and Health Engineering, Key Laboratory for Space Biosciences and Biotechnology, School of Life Sciences, Northwestern Polytechnical University, No. 127 Youyi West Road, Beilin District, Xi’an 710072, China; fanxiaolinzi@163.com; 2 Xi’an Key Laboratory of Toxicology and Biological Effect, Institute for Hygiene of Ordnance Industry, Xi’an 710065, China

**Keywords:** blast traumatic brain injury, 5-HT reuptake inhibitors, depressive-like behaviors, brain-derived neurotrophic factor

## Abstract

Background: Blast traumatic brain injury (bTBI) can result in depression-like behaviors in the acute and chronic phases. SSRIs have been shown to significantly alleviate depression-like behaviors in animal models of traumatic brain injury (TBI) by increasing serotonin (5-HT) and brain-derived neurotrophic factor (BDNF) in the hippocampus. However, the therapeutic effects of SSRIs on depression caused by bTBI remain unclear. Objective: Therefore, this study was aimed at investigating the therapeutic effects of SSRIs on depression-like behaviors in bTBI models. Methods: We created a rat model to study mild TBI by subjecting rats to increased blast overpressures (BOP) and injecting fluoxetine and escitalopram SSRIs intraperitoneally for 28 days. Results: On day 14 post-BOP exposure, rats treated with SSRIs showed decreased depression-like behaviors. This finding was accompanied by higher 5-HT levels in the hippocampus and increased numbers of Nestin-positive cells in the dentate gyrus. Furthermore, rats treated with SSRIs exhibited increased pCREB and BDNF protein expression in the hippocampus on days 7, 14, and 28 after bTBI. Conclusions: Overall, our findings indicate that SSRI-induced recovery from depression-like behaviors after mild bTBI is associated with the upregulation of 5-HT levels, pCREB and BDNF expression, and neurogenesis in the hippocampus.

## 1. Introduction

The bTBI is a traumatic brain injury caused by the direct effects of complex pressure waves generated by explosions [1]. During the wars in Iraq and Afghanistan (2001–2011), approximately 400,000 members of the U.S. Armed Forces suffered at least one traumatic brain injury during service. Nearly two-thirds of these soldiers experienced explosions, and approximately 83.3% of those who experienced an explosion suffered minor, uncomplicated bTBI or concussion [2,3,4]. Many soldiers who suffered mild bTBI in the global war on terror return home because of psychiatric disorders including anxiety and depression [4,5,6]. Posttraumatic stress disorder (PTSD) and depression are two frequent psychological issues that individuals encounter after TBI. Previous studies have indicated that individuals exhibit different patterns of co-occurrence between PTSD and depressive symptoms after experiencing trauma, including low symptoms, PTSD-dominant symptoms, depression-dominant symptoms, and PTSD combined with depressive symptoms [7].

The primary focus of white matter damage caused by TBI is on traumatic axonal injury [8]. Long-distance axons in white matter tracts can be damaged by twisting, stretching, and compression [9]. The characteristic feature is the dispersed distribution of damaged axons within the white matter [10]. Studies have shown axonal injury in multiple brain regions in TBI patients [11,12]. In recent years, numerous studies have shown that neurogenesis in the dentate gyrus (DG) of the hippocampus lasts 1 month in animal TBI models and sometimes up to 1 year after traumatic brain injury in humans [13]. Long-term antidepressant treatment has been shown to alleviate depression by increasing neurogenesis in the adult rat hippocampus [14].

BDNF, one of the most widely distributed neurotrophic factors in the mammalian brain, plays key roles in neural growth, survival, and plasticity [15,16]. A close relationship between neurogenesis and BDNF has been reported [17,18,19]. The regulation of BDNF expression involves pCREB, which prevents neuronal apoptosis. The long-term use of antidepressants can lead to increased expression of pCREB, thereby exerting therapeutic effects. However, the mechanism underlying the therapeutic effect of pCREB remains unknown [20,21]. Additionally, pCREB has long been considered to play a crucial role in hippocampal neurogenesis after TBI, although its exact mechanisms are currently under-researched [22,23].

SSIRs, drugs that enhance 5-HT and endogenous BDNF expression, may prevent cognitive impairment after TBI by upregulating BDNF expression in the brain [24]. However, whether the early use of SSRIs can effectively treat acute or chronic mild bTBI-induced depressive-like behaviors remains unclear. Here, we propose that the effects of antidepressants are likely to be associated with neurogenesis in bTBI, particularly involving changes in 5-HT, pCREB, and BDNF.

In our study, we created bTBI models in rats by using increasing blast overpressures. We then selected the SSRIs fluoxetine and escitalopram as first-line antidepressants to study the mechanisms of therapeutic effects on depression-like behaviors in bTBI models. Our behavioral examinations indicated that bTBI induced increased depression-like behaviors in rats, as observed in open-field and forced-swimming tests. However, treatment with SSRIs ameliorated depression-like behaviors in bTBI rats. Western blotting, ELISA, and immunofluorescence staining demonstrated that SSRIs increased 5-HT levels, elevated the expressions of pCREB and BDNF, and increased Nestin-positive cells in the hippocampus. In summary, this research reveals the therapeutic effect of SSRI interventions on depressive-like behaviors after bTBI and the possible molecular mechanisms involved, which provides a new exploration of early intervention and the possible pathogenesis of depression-like behaviors after mild bTBI.

## 2. Methods

### 2.1. Animals

**Figure 1:**
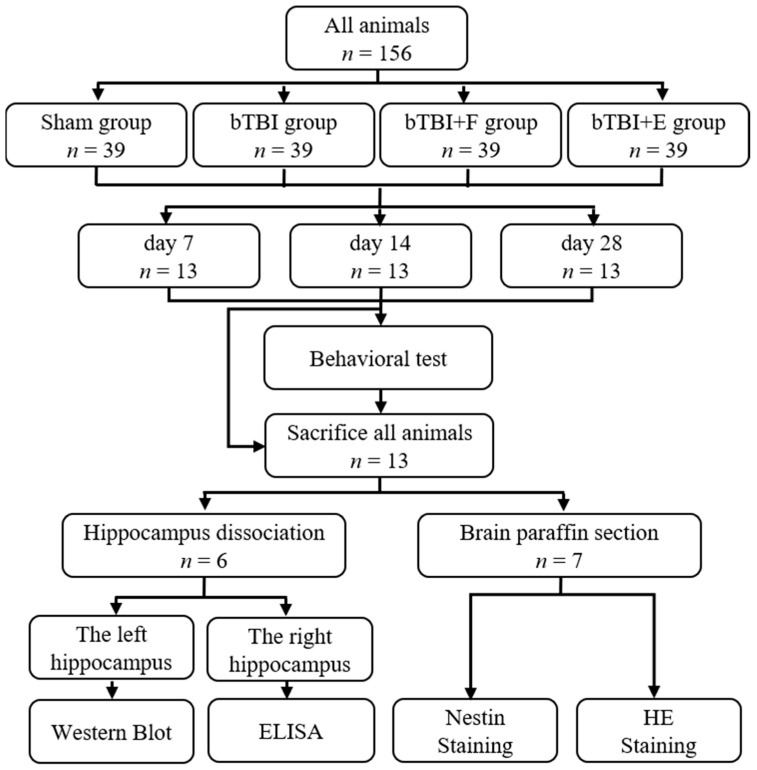
Schematic outline of the experimental schedule.

To avoid interference from the estrous cycle in female animals in behavioral studies, we used male animals to construct the bTBI model. Additionally, because the people who suffer bTBI on the battlefield are primarily male, using male animals to construct the bTBI model aligns closely with actual clinical situations. Male Sprague–Dawley rats (200–220 g) were obtained from Speyford Experimental Animal Science and Technology (Beijing, China). The rats were housed under a 12 h light/dark cycle at 22–24 °C and were given food and water ad libitum. All animals were acclimated for at least 5 days before experiments. The rats were divided into the following four groups: sham, bTBI, bTBI+F (fluoxetine), and bTBI+E (escitalopram). In the sham and bTBI groups (*n* = 39 per group), all animals were intraperitoneally injected with saline. Animals in the bTBI+F and bTBI+E (*n* = 39 per group) groups were intraperitoneally injected with fluoxetine HCl (56296-78-7, purchased from Aladdin Biochemical Technology Co., Ltd., Shanghai, China) and escitalopram (HY-14258, obtained from Med Chem Express (MCE), Shanghai, China) at doses of 10 mg/kg/day bodyweight 7, 14, and 28 days post-BOP exposure, respectively [25] (Figure 1).

In this study, 156 animals were randomly allocated into four groups (sham, bTBI, bTBI+E, and bTBI+F), with 39 animals per group. At three post-modeling time points (days 7, 14, and 28), 13 animals from each group were randomly selected for behavioral and molecular analyses. Among these, 10 animals underwent behavioral testing, after which six were euthanized for hippocampal dissection. The left hippocampus was used for ELISA and the right for Western Blot. The remaining seven brains were processed into paraffin sections for HE staining and immunofluorescence.

### 2.2. Construction of bTBI Models

In this study, we referred to the literature regarding the induction of bTBI in model animals subjected to increased BOP [26]. A schematic illustration of our newly designed shock wave delivery apparatus used in these studies is shown in Supplemental Figure S1. Before blast exposure, the rats were anesthetized with pentobarbital sodium at 60 mg/kg via intraperitoneal injection (Sigma-Aldrich, St. Louis, MO, USA). Rats in the sham group received anesthesia and noise exposure without BOP. The anesthetized animals were placed next to the shock tube. All rats were modeled in a prone position and protected with protective equipment around the body. A single pure primary blast wave (blast overpressure, 100–120 kPa; impact duration, 3–8 ms) was produced by compressed gas shock tubes. As a quality control measure, the pressure sensors used in our experiments were PCB piezotronics (Depew, NY, USA) model 113B28. All data were captured at a sampling rate of 2.0 MHz. The usual duration for data collection in each experiment was 20 ms.

### 2.3. Behavioral Assessment

The open field test (OFT) and forced swimming test (FST) were performed to assess depression-like behaviors in the animals on days 7, 14, and 28 post-BOP exposure [27,28,29,30]. Before each behavior, animals were transferred to the test room at least 2 h before the test.

In the OFT test [27], the animals’ behaviors were recorded with a camera mounted above the arena and were subsequently analyzed in Locomotor Activity/Open Field software V8.02 (TSE Multi Conditioning System, Homburg, Germany). The OFT was divided into two zones: an outside zone (25 cm wide) and a center zone (50 × 50 cm; L × W). During the test, a rat was placed in the center square and its activity was recorded for 5 min. The total distance traveled and time and distance in the center square were recorded and analyzed. After one rat was tested, the OFT unit was cleaned with a 75% alcohol solution before testing the next rat.

In the FST, a hopeless state was represented by immobility [31]. Animals were placed in plastic cylinders (height, 50 cm; diameter, 19 cm) containing water (25 ± 1 °C; depth, 30 cm) on two occasions. These two occasions included a pre-test (15 min) and a test (5 min), and the sessions were conducted 24 h apart. The immobility time over 5 min was evaluated after each rat (*n* = 10 per group) was placed in water by a blinded observer.

### 2.4. Enzyme-Linked Immunosorbent Assay

On days 7, 14, and 28 post-BOP exposure, the rats were deeply anesthetized with pentobarbital sodium (60 mg/kg). Euthanasia was performed through carbon dioxide (CO_2_) inhalation. The left hippocampus was rapidly placed in RIPA solution and subjected to ultrasonic disruption. (HY-K1001; MCE, Shanghai, China) containing protease inhibitors (HY-K0010; MCE, Shanghai, China). Samples were centrifuged at 4 °C and 12,000 rpm for 20 min. The supernatants were collected for enzyme-linked immunosorbent assay (ELISA) analysis. The amounts of 5-HT in the rat hippocampus (*n* = 6 per group) were detected with a rat 5-HT ELISA kit (ml058927, Enzyme-linked Biotechnology Co., Ltd., Shanghai, China), according to the manufacturer’s instructions.

### 2.5. Immunofluorescence Staining

On days 7, 14, and 28 post-BOP exposure, all rats were deeply anesthetized with pentobarbital sodium (60 mg/kg) and then perfused via the left ventricle with PBS solution containing 4% paraformaldehyde (pH 7.4). The brain tissues embedded in paraffin were sliced into consecutive 5 μm sections, then placed in an oven at a constant temperature of 60 °C for 2 h. Antigen retrieval was performed with sodium citrate (Bioss, Beijing, China) in a microwave on moderate power for 15 min. Subsequent 10% goat serum was used to block the sections for 30 min at 37 °C. The sections were then exposed to anti-Nestin (1:200; Abcam, Cambridge, UK; ab254048) primary antibodies overnight at 4 °C. The following day, the sections were rinsed in PBS for 5 min and treated with biotinylated anti-mouse IgG (H + L) and F(ab′)_2_ fragment (Alexa Fluor^®^ 555 conjugate) (1: 500; Cell Signaling, Danvers, MA, USA; 4409) at 37 °C for 30 min, then washed in PBS for 5 min. Nuclei were counterstained with 4′,6-diamino-2-phenylindole (DAPI). Subsequently, the sections were sealed with a fluorescence-quenching agent. Finally, images were captured with a laser scanning confocal fluorescence microscope (Leica; TSC-SP8; Wetzlar, Germany). The count of Nestin-stained cells in the hippocampal DG area was conducted by blinded investigators.

### 2.6. Western Blotting

On days 7, 14, and 28 post-BOP exposure, rats were deeply anesthetized with pentobarbital sodium (60 mg/kg) and euthanized with carbon dioxide (CO_2_) inhalation. The left hippocampus was rapidly placed in RIPA solution and subjected to ultrasonic disruption (HY-K1001, MCE, Shanghai, China) containing protease inhibitors (HY-K0010; MCE, Shanghai, China). Samples were centrifuged at 12,000 rpm for 20 min at 4 °C. Subsequently, protein concentrations were assessed with a BCA reagent (Thermo Fisher Scientific, Waltham, MA, USA; 23235), according to the manufacturer’s guidelines. After protein concentrations were standardized, protein samples of approximately 20 μg were separated via SDS-PAGE and transferred to PVDF membranes (Millipore, Burlington, MA, USA; IPVH00010). The membranes were then blocked with 5% skimmed milk for 1 h at room temperature. The membranes were subsequently exposed to the following primary antibodies overnight at 4 °C: anti-mature BDNF (1:1000; Abcam, ab226843), anti-CERB (1:1000, Abcam, ab32515), anti-pCREB (1:1000, Abcam, ab254107), and anti-β-actin (1:3000, Abcam, ab8226). Subsequently, the membranes were washed with TBST and incubated with horseradish peroxidase-conjugated secondary antibody (1:4000) for 2 h at room temperature. The membranes were washed with TBST again, and the antibody reactive bands were visualized with an enhanced chemiluminescence detection reagent. Finally, development was conducted with Super Signal chemiluminescence substrate, and the outcomes were evaluated in Image-Pro Plus 6.0.

### 2.7. Statistical Analysis

SPSS (20.0) software was used to analyze the experimental data, and all outcome measures were analyzed with one-way ANOVA. All data are presented as mean ± SEM, and *p* ≤ 0.05 was considered statistically significant.

## 3. Results

### 3.1. bTBI Leads to Neuronal Damage in the DG Region of the Hippocampus on Day 28

**Figure 2:**
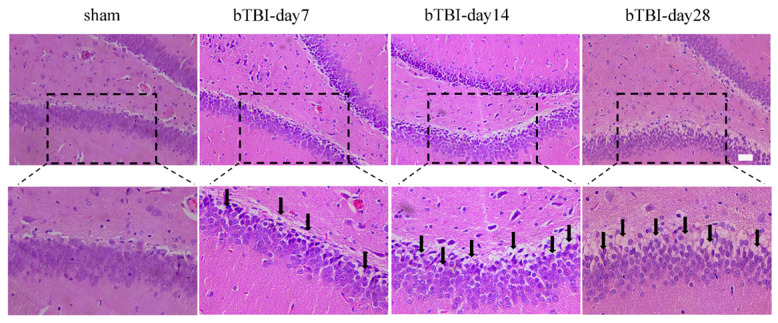
Neuronal damage in the DG region of the hippocampus in bTBI rats on day 28. Representative HE staining image of the hippocampal DG region in TBI group rats. Black arrows indicate abnormal cell morphology. Scale bar = 50 μm.

On day 28 after BOP exposure, we assessed the damage in hippocampal DG neurons caused by bTBI exposure through HE staining. The HE results indicated that the neurons in the hippocampal DG region of the rats in the sham group showed a neat, close arrangement. However, after exposure to bTBI, the neurons in the hippocampal DG region were arranged in a disordered manner, with inconsistent morphology (Figure 2).

### 3.2. SSRIs Alleviate Depressive-like Behaviors in Rats Exposed to bTBI in Behavioral Assessments

**Figure 3:**
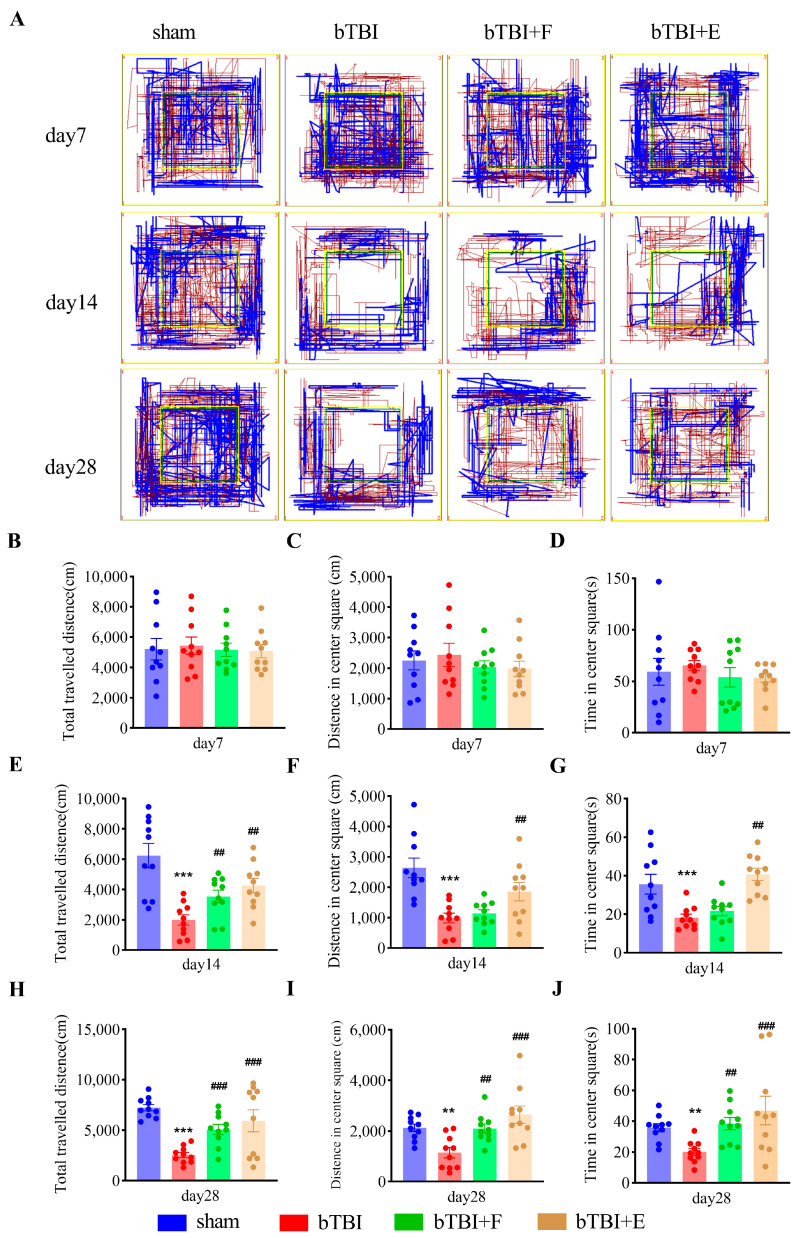
SSRIs treatment reverses depressive-like behaviors in bTBI rats on days 14 and 28 post-BOP exposure in OFT. (**A**) Typical animal movement track. Blue lines indicate the movement trajectories of the animal standing upright, and brown lines indicate the animal’s crawling tracks. (**B**–**J**) Traveled distance (**B**,**E**,**H**), distance in the center square (**C**,**F**,**I**), and time in the center square (**D**,**G**,**J**) of the OFT on days 7 (**B**–**D**), 14 (**E**–**G**) and 28 (**H**–**J**). Data are presented as mean ± SEM, *n* = 10 per group; ** *p* < 0.01, *** *p* < 0.001 versus sham group; ## *p* < 0.05, ### *p* < 0.001 versus bTBI group, as determined by one-way ANOVA.

The open-field test was performed to assess the locomotor activity and depressive-like behaviors in rodents. In the OFT, rats in the bTBI group showed increased depressive-like behaviors on days 14 and 28 post-BOP exposure, which were alleviated by SSRI treatment (Figure 3A). On day 7 post-BOP exposure, no significant difference was observed in the total traveled distance (F_(3,36)_ = 0.84; *p* > 0.05), center square distance (F_(3,36)_ = 0.50; *p* > 0.05), and time (F_(3,36)_ = 0.42; *p* > 0.05) in the OFT among the four groups (Figure 3B–D). However, on day 14, animals in the bTBI group demonstrated significantly lower total traveled distance (F_(3,36)_ = 10.65; *p* < 0.001), center square distance (F_(3,36)_ = 9.89; *p* < 0.001), and time (F_(3,36)_ = 10.10; *p* < 0.001) than animals in the sham (*p* ≤ 0.05) and bTBI+E (*p* ≤ 0.05) groups (Figure 3E–G). Analysis of the recordings on day 28 further confirmed the antidepressant effects of SSRIs and indicated better therapeutic effects of escitalopram than fluoxetine. Compared with the bTBI group, rats in the bTBI+F (*p* ≤ 0.05) and bTBI+E (*p* ≤ 0.05) groups displayed significantly greater total traveled distance (F_(3,36)_ = 9.72; *p* ≤ 0.001), center square distance (F_(3,36)_ = 7.36; *p* ≤ 0.001), and time (F_(3,36)_ = 4.44; *p* ≤ 0.01). No significant difference between the bTBI+F and bTBI+E groups was observed (Figure 3H–J). Together, these results indicated that bTBI caused severe depression-like behaviors in rats on days 14 and 28 post-BOP exposure. However, treatment with SSRIs significantly ameliorated these behaviors.

**Figure 4:**
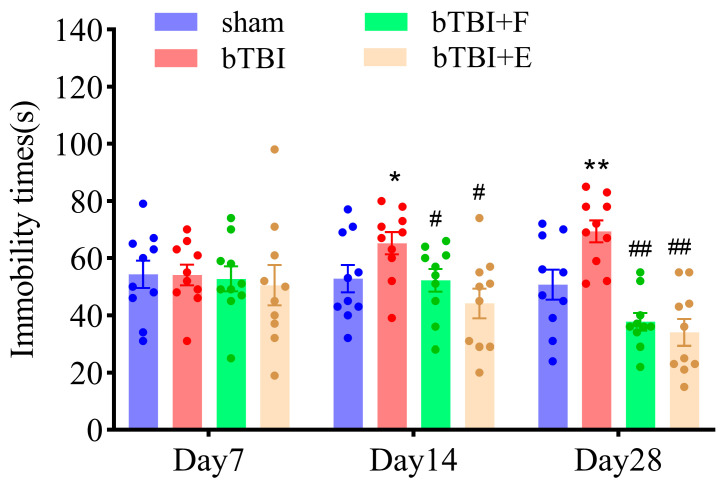
SSRIs significantly decrease the immobility time of bTBI rats on days 14 and 28 after bTBI. Data are presented as mean ± SEM, *n* = 10 per group; * *p* < 0.05, ** *p* < 0.01, versus sham group. # *p* < 0.05, ## *p* < 0.01, versus bTBI group, as determined by one-way ANOVA.

The FST was performed to assess the level of hopelessness and detect depression-like behavior in the rats. A state of hopelessness was represented by immobility [32]. In the FST, animals in the bTBI group had significantly longer immobility times than observed in the sham, bTBI+F, and bTBI+E groups on days 14 (F_(3,36)_ = 3.75; *p* ≤ 0.05) and 28 (F_(3,36)_ = 14.02; *p* ≤ 0.001). No significant difference between the bTBI+F and bTBI+E groups was observed (Figure 4). These findings indicated that SSRIs ameliorated the depression-like behaviors caused by bTBI in the animals.

### 3.3. SSRIs Restore Diminished 5-HT Levels in the Hippocampus in Rats Exposed to bTBI

**Figure 5:**
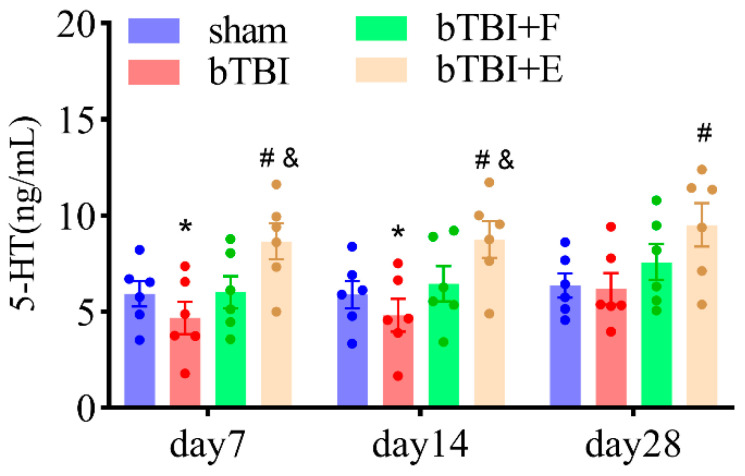
Increased serotonin levels in bTBI animals treated with escitalopram. Data are presented as mean ± SEM, *n* = 6 per group; * *p* < 0.05 versus sham group; # *p* < 0.05 versus bTBI group; & *p* < 0.05 versus bTBI+F group, as determined by one-way ANOVA.

On day 7 post-BOP exposure, the 5-HT levels in the hippocampus were 5.9 ± 0.6, 3.7 ± 0.4, 4.8 ± 0.4, and 7.0 ± 0.6 ng/mL in the sham, bTBI, bTBI+F, and bTBI+E groups, respectively (*n* = 6 per group). Compared with those in the bTBI and bTBI+F groups, the levels of 5-HT were significantly higher in the bTBI+E group (F_(3,20)_ = 6.82; *p* ≤ 0.01). Significant differences between sham and bTBI groups were observed (*p* ≤ 0.05). On day 14 post-BOP exposure, the 5-HT levels in the hippocampus were 5.9 ± 0.7, 4.6 ± 0.9, 6.0 ± 0.9, and 8.7 ± 1.0 ng/mL in the sham, bTBI, bTBI+F, and bTBI+E groups, respectively (*n* = 6 per group). Compared with those in the bTBI and bTBI+F groups, the levels of 5-HT were significantly higher in the bTBI+E group (F_(3,20)_ = 4.13; *p* ≤ 0.01). Additionally, the 5-HT levels in the sham group were significantly lower than those in the bTBI group (*p* ≤ 0.05). On day 28 post-BOP exposure, the 5-HT levels in the hippocampus were 6.4 ± 0.6, 6.2 ± 0.8, 7.6 ± 0.9, and 10.2 ± 0.8 ng/mL in the sham, bTBI, bTBI+F, and bTBI+E groups, respectively (n = 6 per group). Compared with those in the bTBI group, the levels of 5-HT were significantly higher in the bTBI+E group (F_(3,20)_ = 5.28; *p* ≤ 0.01). However, 5-HT levels were comparable between the sham and bTBI groups. (Figure 5).

### 3.4. Rats Show More Neurons Positive for Nestin in the Hippocampal DG Area After SSRI Treatment

**Figure 6:**
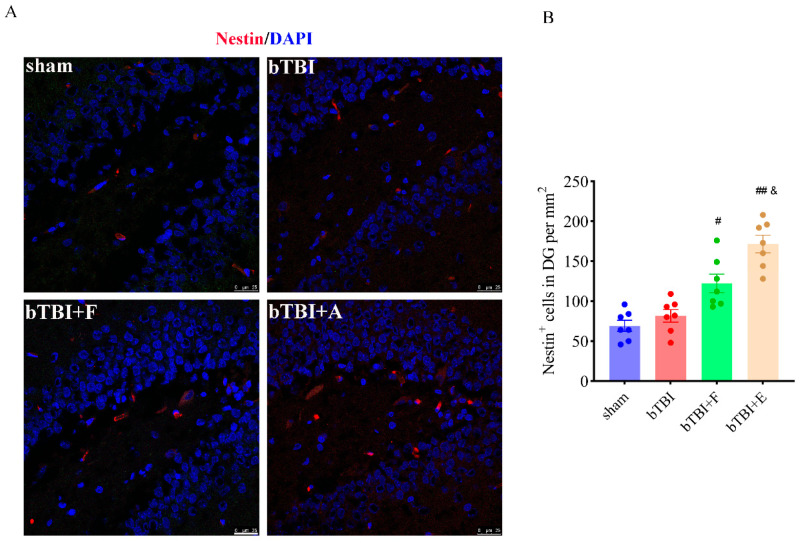
Treatment with escitalopram increases the number of Nestin-positive cells in the hippocampal DG area. (**A**) Representative images of Nestin immunofluorescence staining in the hippocampal DG area in animals in each group on day 28 after bTBI. Blue = DAPI. Red = Nestin. (**B**) Quantification of the neural stem cell marker Nestin in the hippocampal DG area. Data are presented as mean ± SEM, *n* = 7 per group; # *p* < 0.05, ## *p* < 0.01 versus bTBI group; & *p* < 0.05 versus bTBI+F group, as determined by one-way ANOVA. Scale bar = 25 µm.

In recent years, brain damage has been found to lead to the re-expression of Nestin in the rat brain, thus suggesting that increased expression of Nestin under pathological conditions may be associated with the recovery of glial cell function and structural reorganization [33,34]. We compared cells stained with Nestin in the hippocampal DG on days 7, 14, and 28 post-BOP exposure. However, on days 7 and 14 after bTBI, we found almost no Nestin-positive cells in the hippocampal DG area in bTBI and SSRI-treated animals (Supplemental Figure S2). In immunofluorescence staining, cells stained with DAPI appeared blue, whereas those stained with Nestin appeared red (Figure 6A). The number of cells stained with Nestin in DG area per mm^2^ was 68.57 ± 6.73, 84.57 ± 9.05, 121.1 ± 11.5, and 171.4 ± 10.88 in the sham, bTBI, bTBI+F, and bTBI+E groups (*n* = 7 per group). The number of Nestin-positive cells (F_(3,24)_ = 22.06; *p* ≤ 0.001) in the bTBI+F (*p* ≤ 0.05) and bTBI+E (*p* ≤ 0.01) groups was apparently greater than that in the bTBI group. The number of Nestin-positive cells was comparable between the sham and bTBI groups (Figure 6B).

### 3.5. The pCREB/BDNF Signaling Pathway Is Involved in the Effects of SSRIs in the Hippocampus

**Figure 7:**
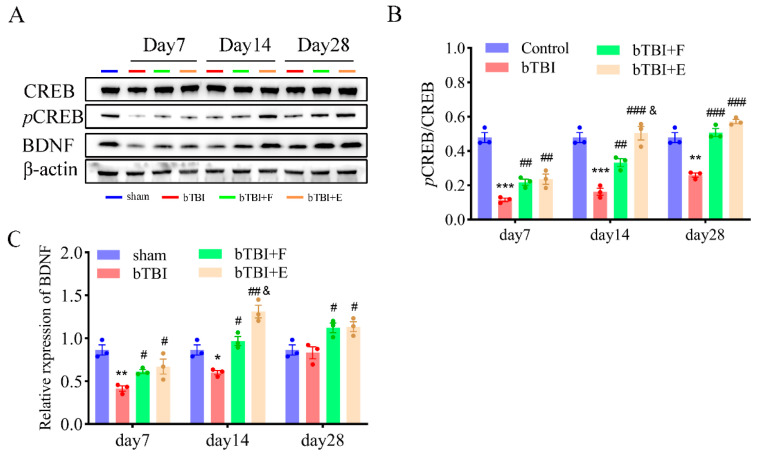
SSRIs markedly increased expression of pCREB and mature BDNF in the hippocampus on days 7, 14, and 28 after bTBI. (**A**) Representative images of Western blots, showing all bands. Quantification of pCREB (**B**) and BDNF (**C**). Data are presented as mean ± SEM, *n* = 3 per group; * *p* < 0.05, ** *p* < 0.01, *** *p* < 0.001 versus sham group; # *p* < 0.05, ## *p* < 0.01, ### *p* < 0.001 versus bTBI group; & *p* < 0.05 versus bTBI+F group, as determined by one-way ANOVA.

We investigated the activation of the pCREB/BDNF signaling pathway in the hippocampus on days 7, 14, and 28 post-BOP exposure (Figure 7A). The bTBI induced markedly lower pCREB expression on days 7 (F_(3,8)_ = 45.16, *p* ≤ 0.001), 14 (F_3,8_ = 29.43; *p* ≤ 0.001), and 28 (F_(3,8)_ = 44.67; *p* ≤ 0.001) than observed in the sham group (day 7: *p* ≤ 0.001, day 14: *p* ≤ 0.001, day 28: *p* ≤ 0.01). Meanwhile, rats in the bTBI group showed lower expression of BDNF on days 7 (F_(3,8)_ = 10.84; *p* ≤ 0.01) and 14 (F_(3,8)_ = 28.32; *p* ≤ 0.001) than observed in the sham group (day 14: *p* ≤ 0.01, day 28: *p* ≤ 0.05). However, SSRIs, particularly escitalopram, resulted in greater expression of pCREB and BDNF on days 7 (pCREB: *p* ≤ 0.01; BDNF: *p* ≤ 0.05), 14 (pCREB: *p* ≤ 0.001; BDNF: *p* ≤ 0.01), and 28 (pCREB: *p* ≤ 0.001; BDNF: *p* ≤ 0.05) than that observed in the bTBI group (Figure 7B,C).

## 4. Discussion

Although substantial research has examined the mechanisms of bTBI (traumatic brain injury), we focused primarily on the changes in brain function after bTBI and treatment. Our study explored the therapeutic effects of SSRIs on depressive-like behaviors in both the acute and chronic phases of bTBI, thereby providing new directions for the clinical use of SSRIs in the treatment of patients with bTBI.

The bTBI model constructed herein is in line with real-world scenarios in actual warfare; we focused solely on blast-induced brain injury, thereby demonstrating the damaging effects of blast waves on the brain. The main factor causing injuries in bTBI was shockwaves, whereas factors contributing to injuries in TBI included impact and penetration. In warfare, blast-induced traumatic brain injury is the primary type of traumatic brain injury. The mechanisms, patterns, and severity of brain injuries differ between blast-related and non-blast-related injuries [4]. The acute clinical manifestations of blast-related mild TBI are the prevalence of post-concussion syndrome, post-traumatic stress disorder, depression, and chronic pain, which are elevated following blast-related mild TBI [35].

SSRIs selectively inhibit the reuptake of 5-HT, thus indirectly increasing the concentrations of 5-HT in the synaptic cleft, enhancing the levels of 5-HT in the brain, and acting as antidepressants. Under the action of the 5-HT transporter and monoamine oxidase, 5-HT is recycled, thereby decreasing extracellular 5-HT levels. During this reuptake process, some 5-HT is absorbed by cells. In some studies, levels of 5-HT have been measured in homogenized brain tissue [36]. Studies have indicated their effectiveness in enhancing working memory [37,38]. Moreover, 5-HT plays a crucial role in maintaining nervous system stability and mood regulation, and its levels are inversely associated with depression severity [39]. The monoamine hypothesis suggests that 5-HT deficiency is a major cause of depression [40]. Therefore, fluoxetine and escitalopram were selected as SSRIs for this research. Studies have shown that although fluoxetine and escitalopram differ in their onset time, they yield consistent outcomes. One explanation for this difference is escitalopram’s faster effects on nerve regeneration in the hippocampus, thus ultimately ameliorating depressive symptoms through relevant pathways [41].

In this research, we focused on the effects of SSRIs on the behaviors of rats on days 7, 14, and 28 after BOP exposure. The administration of escitalopram and fluoxetine significantly alleviated depression-like behaviors on days 14 and 28 after BOP exposure. However, escitalopram had better therapeutic effects than fluoxetine, such as more time spent in the center square in the OFT and less immobility in the FST. Additionally, a notable rise in the 5-HT concentrations in the hippocampus in the bTBI+E group was observed on days 7, 14, and 28 post-BOP exposure. These findings suggested that treatment with SSRIs before behavioral abnormalities after bTBI might be a viable approach to avert depression in both the acute and chronic phases of mild bTBI.

Interestingly, we observed a mismatch between the levels of 5-HT and BDNF and the behavioral alterations after the trauma. No behavioral alterations were observed at day 7 in the bTBI group, whereas a strong decrease in the biochemical parameters was observed at the same time point. In contrast, whereas no difference was observed in serotonin or BDNF levels between the sham and bTBI groups at day 28, the behavioral alterations in the bTBI group were significant at this time point. The changes in biochemical parameters within the body might not necessarily lead to significant behavioral changes in the short term. For example, the elevation of monoamines in the synaptic cleft occurs within a short period of time after the administration of an antidepressant drug. Despite this rapid elevation in monoamines from the first administration, typical antidepressants require weeks to months of chronic use to exert their therapeutic effects [36].

Recent studies have provided strong evidence that the loss of neurons is a key factor in depressive disorders, and antidepressant treatment increases the expression of BDNF in the granule layer of the hippocampal DG, thereby contributing to the regulation of stress and antidepressant neurogenesis [17]. Therefore, we focused on neurogenesis in the DG area of the hippocampus as a potential mechanism underlying recuperation from depression-like actions caused by bTBI on day 28 post-BOP exposure. Treatment with escitalopram increased the number of Nestin-positive cells in the hippocampal DG area in the bTBI+E group on day 28 after BOP exposure. Nestin is an intermediate filament protein whose expression is closely associated with the proliferation and differentiation of neural stem cells (NSCs). This protein serves as a characteristic marker of NSCs, and its expression rapidly decreases or disappears after birth. NSCs repair damage to the nervous system, regulate neurogenesis, and are significantly associated with emotions, learning, and memory [42,43]. These findings suggested that treatment with SSRI escitalopram activated neurogenesis in the hippocampus and were also consistent with previous reports indicating that BDNF mediated neuroplasticity [44,45].

CREB has a protective effect on neurons, and activated CREB (pCREB) promotes the neural differentiation of animal pluripotent stem cells and protects neural function [46]. However, after brain injury, the expression of CREB is suppressed and neuronal apoptosis is accelerated [47]. Under bTBI conditions, the decreased basal level of pCREB lasts for 28 days in the hippocampal DG area [44]. BDNF is a downstream factor regulated by CREB, and activation of the CREB/BDNF signaling pathway has been found to decrease hippocampal neuron damage in rats with depression [46,48]. The antidepressant effects of drugs are also associated with activation of the CREB/BDNF signaling pathway in mice with depression [49]. Similarly, we observed that the levels of pCREB and BDNF were significantly diminished in the hippocampus in the bTBI rats. This result suggested that BOP exposure might decrease the expression of pCREB and BDNF. In contrast, the protein expression of pCREB and BDNF in the bTBI+E and bTBI+F groups was significantly greater than that in the bTBI group. Therefore, SSRI intervention therapy might restrict the downregulation of pCREB in the animal models constructed in this study. Treatment with SSRIs resulted in notable increases in the levels of BDNF and 5-HT in the extracellular space. The increased 5-HT and BDNF might also play important roles in the downregulation of pCREB. Nonetheless, the link between 5-HT and the downregulation of pCREB remains ambiguous.

Research has suggested associations between depression and dysfunction in multiple brain regions [50]. In this study, we focused on the hippocampus, because of its well-established role in neurogenesis after TBI [13]. Nevertheless, the entire brain is affected by bTBI model induction and SSRI treatment, thus suggesting a need to investigate neurogenesis in various brain regions. Additionally, we considered only the effects of SSRI treatments on depression-like behaviors in rats post-BOP exposure, overlooking other deficits such as anxiety and cognitive dysfunction.

## 5. Conclusions

In conclusion, our findings indicate that SSRI-induced recovery from depression-like behaviors after mild bTBI is associated with the upregulation of 5-HT levels, pCREB and BDNF expression, and neurogenesis in the hippocampus.

## Data Availability

All data included in this study are available upon request via contact with the corresponding author (Junhong Gao, gaoxing2285@126.com). The data are not publicly available due to privacy.

## Supplementary Materials

The following supporting information can be downloaded at: https://www.mdpi.com/article/10.3390/brainsci15030236/s1, Figure S1: Illustration of the newly designed shock wave delivery apparatus used in this study; Figure S2: Immunofluorescence Staining of nestin.
