# Supplementary material for: The pCREB/BDNF Pathway in the Hippocampus Is Involved in the Therapeutic Effect of Selective 5-HT Reuptake Inhibitors in Adult Male Rats Exposed to Blast Traumatic Brain Injury
Source: Brain Sci. 2025 Feb 24;15(3):236. doi: 10.3390/brainsci15030236 (PMC11940387; doi:10.3390/brainsci15030236)
Supplement: Supplementary file 1 [file brainsci-15-00236-s001.zip › brainsci-3473109-supplementary.pdf]

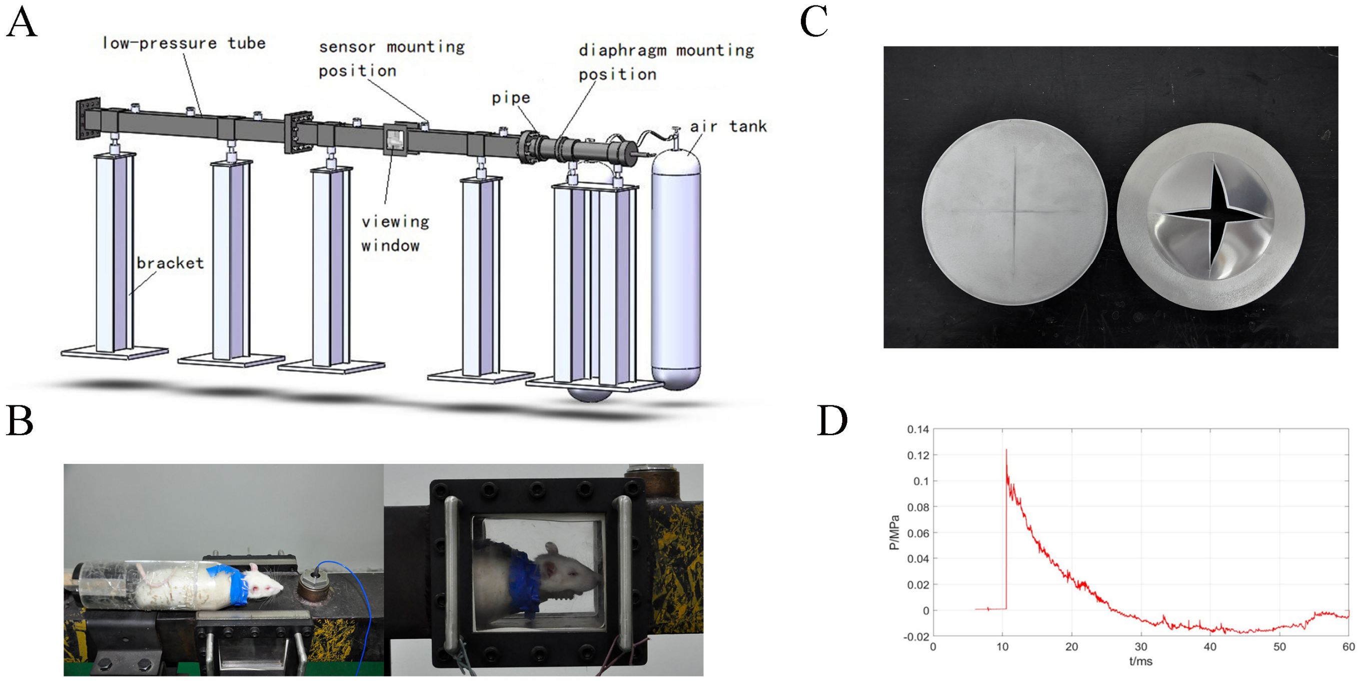

Figure S1. Illustration of the newly designed shock wave delivery apparatus used in this study. (A) Schematic illustration. (B) Aluminum diaphragm with cross groove. (C) Animal wrapped in a harness to minimize head and body motion during blast exposure. (D) Representative overpressure profile.

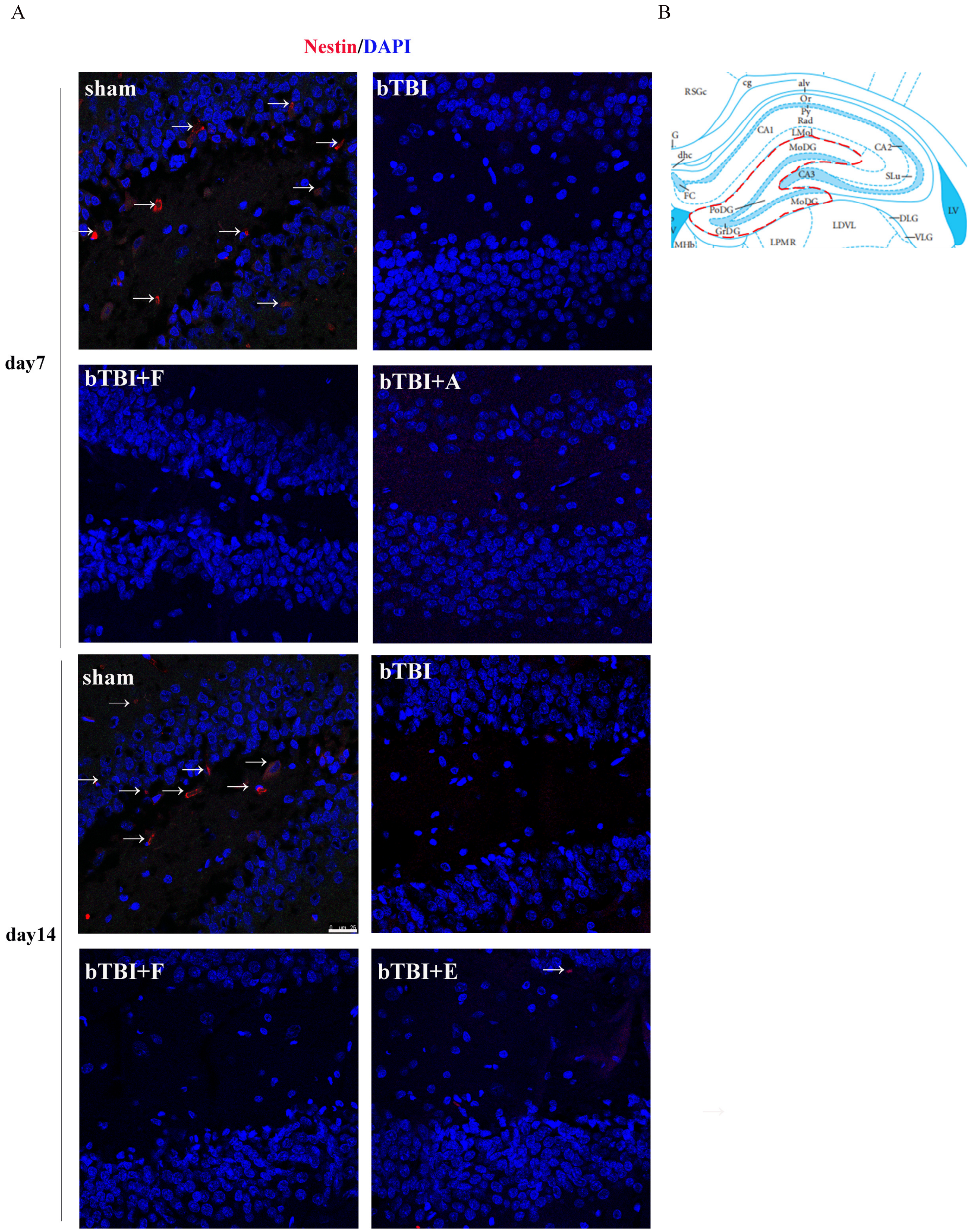

Figure S2. Immunofluorescence Staining of nestin. (A) Nestin staining in the DG area of hippocampus on day 7 and day 14 after bTBI exposure. Blue = DAPI. Red = Nestin. Scale bar = 25 μm. (B) Illustration of the border of the gyrus dentate.
